# AWSCS-A System to Evaluate Different Approaches for the Automatic Composition and Execution of Web Services Flows

**DOI:** 10.1371/journal.pone.0127677

**Published:** 2015-06-12

**Authors:** Bruno Tardiole Kuehne, Julio Cezar Estrella, Luiz Henrique Nunes, Edvard Martins de Oliveira, Luis Hideo Nakamura, Carlos Henrique Gomes Ferreira, Regina Helena Carlucci Santana, Stephan Reiff-Marganiec, Marcos José Santana

**Affiliations:** 1 IESTI, Federal University of Itajubá, Itajubá, MG, Brazil; 2 ICMC, University of São Paulo, São Carlos, SP, Brazil; 3 Department of Computer Science, University of Leicester, Leicester, UK; King Saud University, Kingdom of Saudi Arabia, SAUDI ARABIA

## Abstract

This paper proposes a system named AWSCS (Automatic Web Service Composition System) to evaluate different approaches for automatic composition of Web services, based on QoS parameters that are measured at execution time. The AWSCS is a system to implement different approaches for automatic composition of Web services and also to execute the resulting flows from these approaches. Aiming at demonstrating the results of this paper, a scenario was developed, where empirical flows were built to demonstrate the operation of AWSCS, since algorithms for automatic composition are not readily available to test. The results allow us to study the behaviour of running composite Web services, when flows with the same functionality but different problem-solving strategies were compared. Furthermore, we observed that the influence of the load applied on the running system as the type of load submitted to the system is an important factor to define which approach for the Web service composition can achieve the best performance in production.

## Introduction

Web services have emerged from paradigm of distributed computing called Service-Oriented Computing (SOC). This enabled enterprises to integrate services across different platforms and technologies through the composition of services [1].

The development of composite Web services is a complex task for developers, possibly because the search for Web services which can be part of the Composite Web service is not trivial. In [2] an experiment using students to develop composite Web services is presented. This experiment indicates that the most difficult part of the creation of new composite services is the discovery of pertinent Web services to achieve the goal.

In order to solve this problem the use of semantic Web services was proposed. With semantic Web services it is possible to find services and their relation according to the semantic links between them. It enables the use of services in cooperation for a wider purpose. Semantic Web services enable the Automatic Web Service Composition (AWSC) through algorithms that can find services and compose them, according to the users input and output formatted as semantic rules.

Several proposals of how to achieve automatic Web Services composition are available in the literature. However, the development of AWSC algorithms is a very complex task and their evaluation are usually restricted to a specific environment. Also, the evaluation of the results of these algorithms only considers the semantic quality [3] of the composition, and is based on static Quality of Service (QoS) parameters.

The main language for Web service composition is WS-BPEL. WS-BPEL provides a language for business processes. It extends the Web service interaction model and enables it to support business transactions [4]. WS-BPEL is the standard for Web service composition, being a robust and powerful language to create interaction between Web services. To create a WS-BPEL composite service it is necessary to write the WS-BPEL source code, describe the partner services, compile the code and deploy it in a WS-BPEL engine application server. These tasks make the process of creating a composite service complex, but were crucially manual. However, an AWSC algorithm does generate very make so complex flows and it does not need robust and complex controls of execution as WS-BPEL provides.

For this issue, a lightweight engine that is capable to manage the Composite Web service execution is proposed. AWSC algorithms are restricted to connect the output from one service to the input of another one. The engine proposed in this paper can handle those kind of operations including parallel execution and synchronization.

In this paper we propose a *Automatic Web Service Composition System* (AWSCS), which provides a specific environment designed to evaluate Web services automatic composition approaches under specific conditions. The main goal of AWSCS is to assist decisions through the performance evaluation of the available composition approaches. The evaluation is based on QoS parameters wich are meansuring during the execution, such as response time or availability. A case study using the AWSCS prototype considers some empirical synthetic composition flows. Each one of these flows has the same semantic quality and uses several abstract services to solve the proposed problems. In this scenario, two flows were developed aiming to solve a problem of trip planning.

The paper is organised as follows: Section 1 describes the state of the Art. Section 2 describes the prototype of the AWSCS system proposed in this paper. Section 3 presents an environment used to deploy AWSCS and to run experiments. The results collected are discussed in Section 4. Finally, the conclusions and directions for future work are presented in Section 5.

## 1 Related Work

The terms manual, automatic, static or dynamic composition are common terms in the related literature. According to the papers [5] [6] dynamic and static are classifications that can be applied in automatic and manual composition. Static composition refers to the association of each service implementation at design time. It means that the service to perform each function of the flow is selected by the programmer in design time. In dynamic composition the services instances are selected at runtime, but the kind of service that composes the flow is selected at design time. Manual and automatic composition are two major classifications: in manual composition a graph describing execution flow and logical reference to Web services is planned by a developer. Automatic Web service composition (AWSC) is the process of creation a new composite service according to the request of an user by a computational system without any intervention of humans.

The process of AWSC can be divided in to three main parts: **the matchmaking of the service**, **service selection** and **services execution**. The matchmaking is responsible for the creation of semantic links between services. The semantic link is the connection between services that occurs when one service has an output which is equivalent to the input of another one. An example of matchmaking algorithm is presented in [7], where it checks the full combination of all services available in the registry. The result is a graph with all possibilities of connection between semantic Web services. It was one of the early works in this topic and it has poor performance when there is a large amount of services. It is difficult to calculate all combinations every time the environment changes. To improve the matchmaking other papers also present matchmaking approaches such as in [8], in which the author proposes the development of hybrid Web service matchmaking. In [9] matchmaking framework based on Description Logic for semantic Web service described by RDF4S model is presented. In [10] the author presents the SAWSDL-iMatcher, a matchmaking that can be customised, in which the users can use their preferred strategies for matchmaking according to the application requirements.

After the matchmaking has created all the semantic links between the services, the algorithm for Web service selection is performed to find the most appropriate services to processes the user request. In [11] the matchmaking model for the service composition is made through a multilayer process which creates a plan graph structure within a matrix of semantic links. To make the selection, an Immune-inspired algorithm is used. In [12] the matchmaking algorithm proposed by [7] is used to create the semantic links between the services. Then Dijkstra’s algorithm is applied to find the best path for the service composition. In [13] the author use OWL-S Web service semantic presentation to describe the composition problem as a planning problem described in a standardised way using PDDL. Thus it is possible to use AI techniques to find the best services for the composition. Other approaches for service selection are also presented in [14] [15]. The matchmaking and selection approaches are examples of what would be possible to evaluate using the AWSCS.

Many works have focused on how to automatically compose Web services. Usually, they show algorithms for automatic composition of Web services, but do not present an assessment of the execution of the resultant flow behaviour.

Actually, just a few research works evaluate and compare these proposals. In addition, the assessment of the behaviour of performing in a real environment could not be found in any work in the literature. The evaluation of the performance of Web services composition are presented in [16], [17], [18] and [19]. Zhao proposed a simulation-based environment to make Web services composition based on Petri network models. This work does not evaluate the performance of the automatic composition of Web services.

In [17] an approach of how to choose the best flow through the use of stochastic Petri nets is presented. This work used analytical methods to evaluate the performance of composite service models. So, it is difficult to measure the impact on performance in some specific cases, such as, when the system is overloaded as a whole.

In [18] stochastic Petri nets are also used with the aim of evaluating dynamic manual composition. After the creation of the service flow, the user can manually choose the desired services to use in the service flow, considering their QoS atributes values.

In [19] an approach using queueing Petri nets for modelling Business Process Execution Language (BPEL) flows is also presented. The authors use simulations to evaluate the performance of the composition. In this work they evaluated the change in average response time.

An adaptive genetic programming approach (AGP) for QoS-aware web services composition is proposed in [20]. They merge the services composition and QoS-aware service selection process to meet both functional and non-functional goals. Also, they compared the AGP with a standard genetic algorithm but, as there are no available benchmark datasets for evaluation purpose, they randomly generated five requests with seven sets of web services for the experiments which has the maximum number of 500 web services. The simulation experiments shows the best performance of AGP to discovering a composite service with various workflow constructs that meets the functional requirements as well as QoS requirements.

In [21] is presented the new concept of composition context together with a novel service selection algorithm. Based on the composition context, the authors presented a novel Backward Composition Context based Service Selection (BCCbSS) approach to meet the composition context aware challenges. Although the authors have not implemented these concepts, they are important to study or play real-world scenarios, using the AWSCS.

OWL, OWL-S and SPARQL are used to compose and invoke semantic web services in managing production processes in [22]. The approach involves the combination of three web services, the first one maintains a semantic model that describes the system state, and the others uses the model to compose the domain web services. Unfortunately, the paper does not present any results of a performance evaluation.

In [23] an extensive set of experiments was conducted, using 30 web service repositories (with a total number of 7,275 web services), that are from six simulated predefined composition workflow models. Each workflow corresponds to five web service repositories, that are generated candidate operations for the tasks in the workflow, and also randomly adding operations outside of the workflow. The approach does not depend on a predefined workflow model for QoS-aware service composition problem, wich is better than the IP-based approachs, that cannot ensure global satisfiability. The experiments show that the comprehensive QoS of a composite service found by our approach outperforms that generated by the IP-based available in the literature. The QoS value of a composite dependence graph found is always better than that of a composite service found by the IP-based approach.

We have discusses work in the literature regarding the evaluation of performance in composition Web services. Most of these works do not offer the possibility to evaluate algorithms for automatic composition of Web services. Another point in common between these studies is that they use analytical models or simulation. The problem with using these approaches is that it is very difficult to describe analytically or simulate the execution behaviour of the composition, since several factors may influence the results, such as overloading the system, types of machines used in providers and types of clients that access the system.

## 2 AWSCS

With AWSCS it is possible to make a fair evaluation of automatic composition algorithms, as they are deployed in a common environment, helping the stakeholder to choose the best approach for its solution.

It also allows performance evaluation in a modular way, by inserting monitors that record performance metrics on a log server.

**Fig 1:**
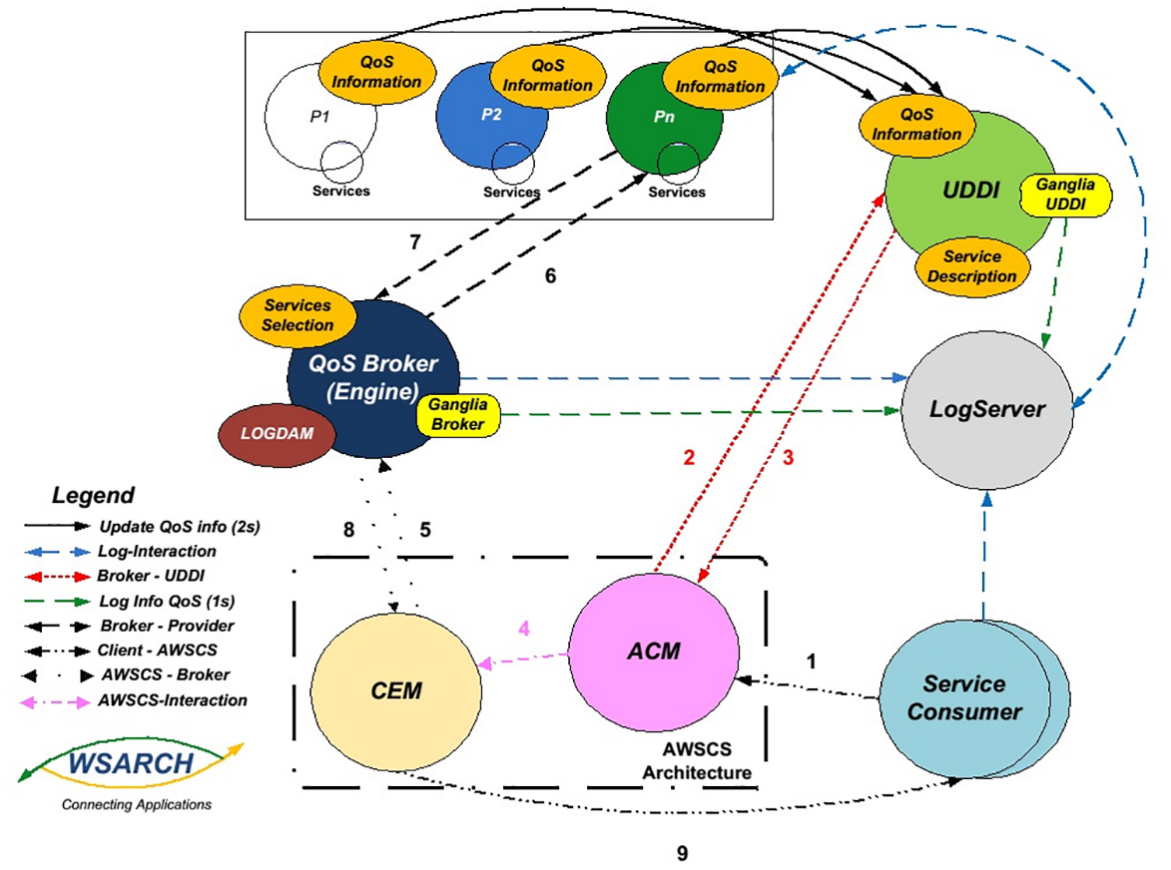
*Web Service Architecture*—WSARCH and AWSCS interaction [24].

The AWSCS architecture has two distinct modules: the automatic composition module (ACM) and the composite execution module (ECM). AWSCS is currently couple to WSARCH which allows for performance evaluation of Web services. The WSARCH architecture [24] has five distinct modules: the client application, the providers, the Broker, the UDDI registry and the Log Server. Fig 1 shows the relationships between these components.

**Fig 2:**
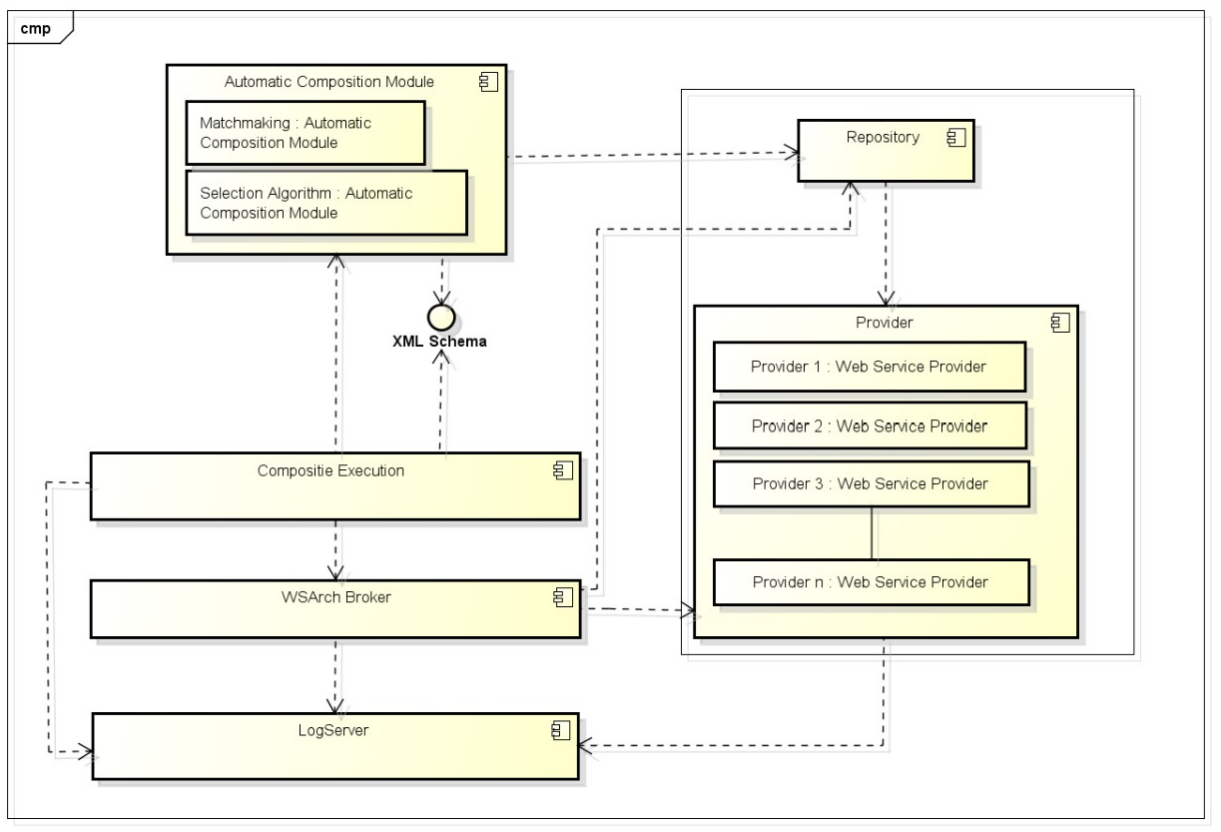
AWSCS Architectural Model.

The component diagram in Fig 2 shows the main modules that compose the architectural model for performance evaluation used by the AWSCS system.

- **Automatic Composition Module**: This module is where the user can deploy the approache for the AWSC. The AWSC approache choose to be used is the responsible to find Web services candidates and generate the flow of execution which fulfil the user request. The prerequisity to make the AWSC approache to work on AWSCS the generation of the output flow following the XML Schema. Since no implementations of algorithms for AWSC are publicly available for download and test, in this paper we simulated this module.
- **Composition Execution Module**: receives the resulting flow from the **Automatic Composition Module**. The CEM is responsible to parse the XML containing the composition flow and control the execution of the composite Web service, ensuring that parallel and sequential services are running in the correct order.
- **Service Provider Repository**: store the semantic descriptions and interfaces to perform the services requests.
- **XML Flow Verifier**: checks the resulting XML flow created by “AWSC Matchmaking + AWSC Selection Algorithm” module. If the XML file is corrupted or poorly formatted, AWSCS will abort the execution.
- **WSArch Broker**: responsible to accomplish the SLA agreement. It looks for a specific service that meets the SLA established between the service and the client module.
- **LogServer**: database which stores all records from execution for later analysis.
- **Web service Providers**: hosts where Web services are deployed and executed.
- **Monitor**: code inserted into WSArch Broker and **AWSCE** modules to measure the time spent on each part of the execution of the service code.

For the functioning of this system, the outcome composite Web service from an AWSC algorithm, must be able to be executed automatically. Using BPEL, it is necessary to assembly files to make a composite service, create partner links and deploy the composite Web service in a composition engine.

In order to allow automatic execution, an XML file containing the description of all Web services needed to perform the composite Web service is generated by the composition algorithm. An XML schema which defines the standard format of AWSCE XML files was proposed.

**Fig 3:**
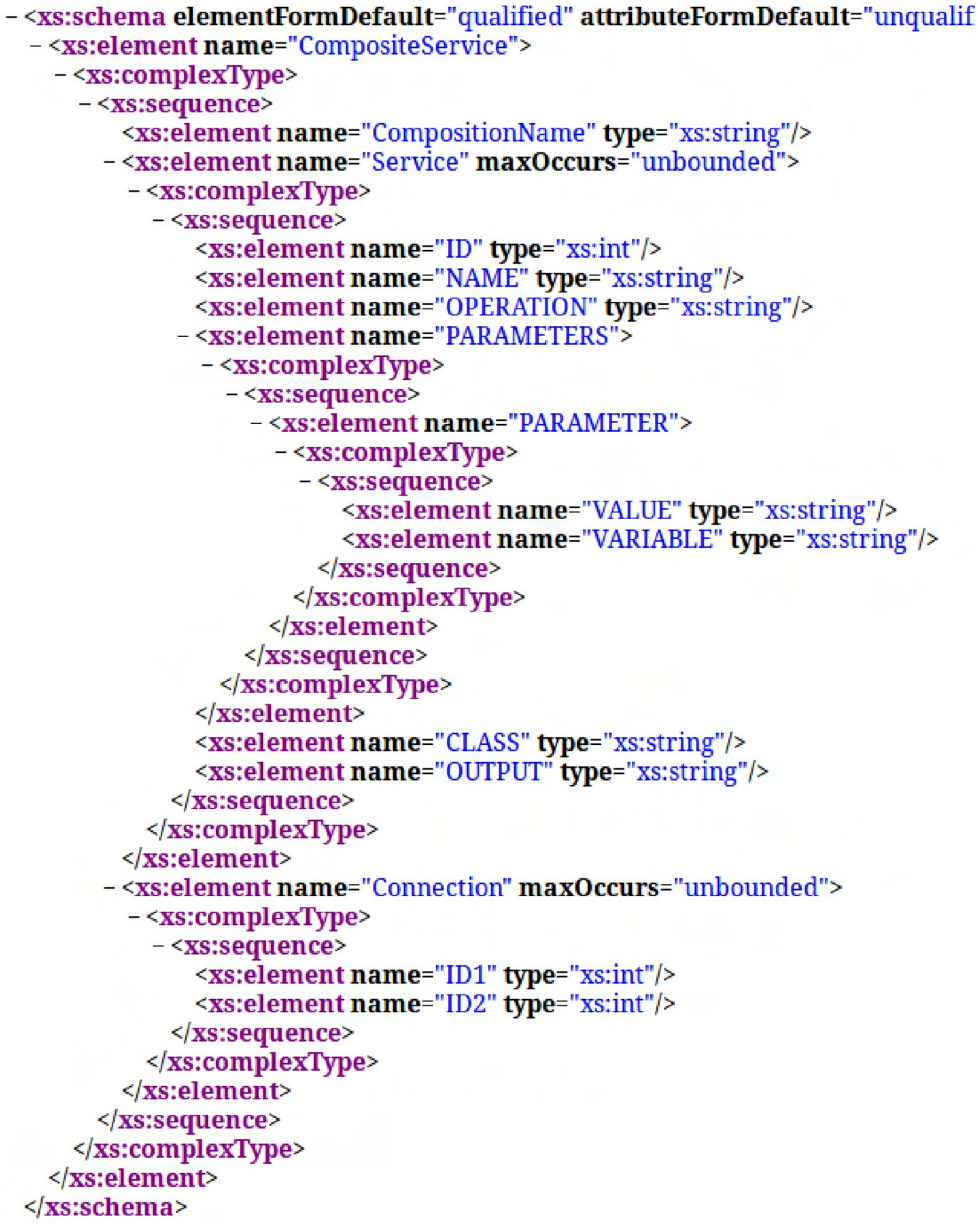
Execution Flow- *XML Schema*.

The XML Schema presented in Fig 3 is composed of three basic elements which are in the **CompositeService** **Tag**: **CompositionName**, **Service** and **Connection**.

- **CompositeService**: Tag that identifies the name of the composition.
- **Service**: For each Web service that is part of the flow, a tag Service is created. This tag contains the basic information to run the Web service. It is composed of 5 elements:
  - **ID**: unique identifier for the Web service.
  - **Name**: The name of Web service;
  - **Operation**: It is the operation name that the Web service will execute.
  - **Parameters**: Corresponds to the input parameters of the operation. It is a complex type, since an operation can have zero or more parameters. A parameter can be either a variable containing the result of another Web service or a value specified by the user who requested a composite Web service.
  - **Class**: Service performance class to be executed. The options are Bronze, Silver or Gold.
  - **Output**: Web service execution output.
- **Connection**: This *tag* is responsible for identifying the execution order of the Web services.

## 3 Performance Evaluation

As no algorithm for automatic composition was found for download and test, for the purpose of testing AWSCS we simulated a scenario where we elaborated possible resulting flows from different approaches for automatic Web service composition.

As the focus of this paper is on the framework for comparing approaches we did not look at evaluating the creation of workflows and the simulated scenario allows to overcome the unavailability of composition algorithms while allowing us to perform the needed evaluations. Two flows with the goal of solving a problem of trip planning was considered. The resulting compositions of the flows have the same semantic quality based on the user request, but different abstract Web services to solve the problem.

For planning and execution of experiments PEESOS (Planning and Execution of Experiments in Service-Oriented Systems) was used as an auxiliary tool [25]. PEESOS is a tool focused in capacity planning and execution in SOA. PEESOS instructs the user to conduct a full factorial experiment from a set of default entries and provides a real testbed environment, in which the workload is generated through a collaborative environment.

All the experiments used in this paper were conducted using PEESOS.

### 3.1 Environment Configuration

The environment used in the tests was composed of two clusters, one whose primary function is to host the WSArch and another that is used to host the clients and AWSCS. The specifications of the machines of the cluster are presented in Tables 1 and 2.

**Table 1:** WSArch Configuration *cluster*.

**WSARCH Environment**		
	***Hosts—Broker, LogServer*, UDDI**	**VM (Provider)**
**Processor**	AMD Processor Vishera 4.2 Ghz	2 Virtual Processor
**Memory**	32 GB RAM DDR3 Corsair Vegeance	2 GB RAM
**Disk**	HD 2TB Seagate Sata III 7200RPM	50 GB
**Operantin System**	Ubuntu Server 12.04 64	Ubuntu Server 12.04 64
**Tools**	JDK 1.7, Axis2 1.5 / Tomcat 7.0, Qemu / KVM	JDK 1.7, Axis2 1.5 / Tomcat 7.0

**Table 2:** Cluster Configuration.

**Cluster**	
**Processor**	Intel® Core™2 Quad Processor Q9400
**Memory**	8 GB RAM DDR3 Kingston
**Disk**	HD 160GB Seagate Sata II 7200RPM
**Operating System**	Linux Ubuntu Server 12.04 64 Bits LTS
**Tools**	Java JDK 1.7

**Fig 4:**
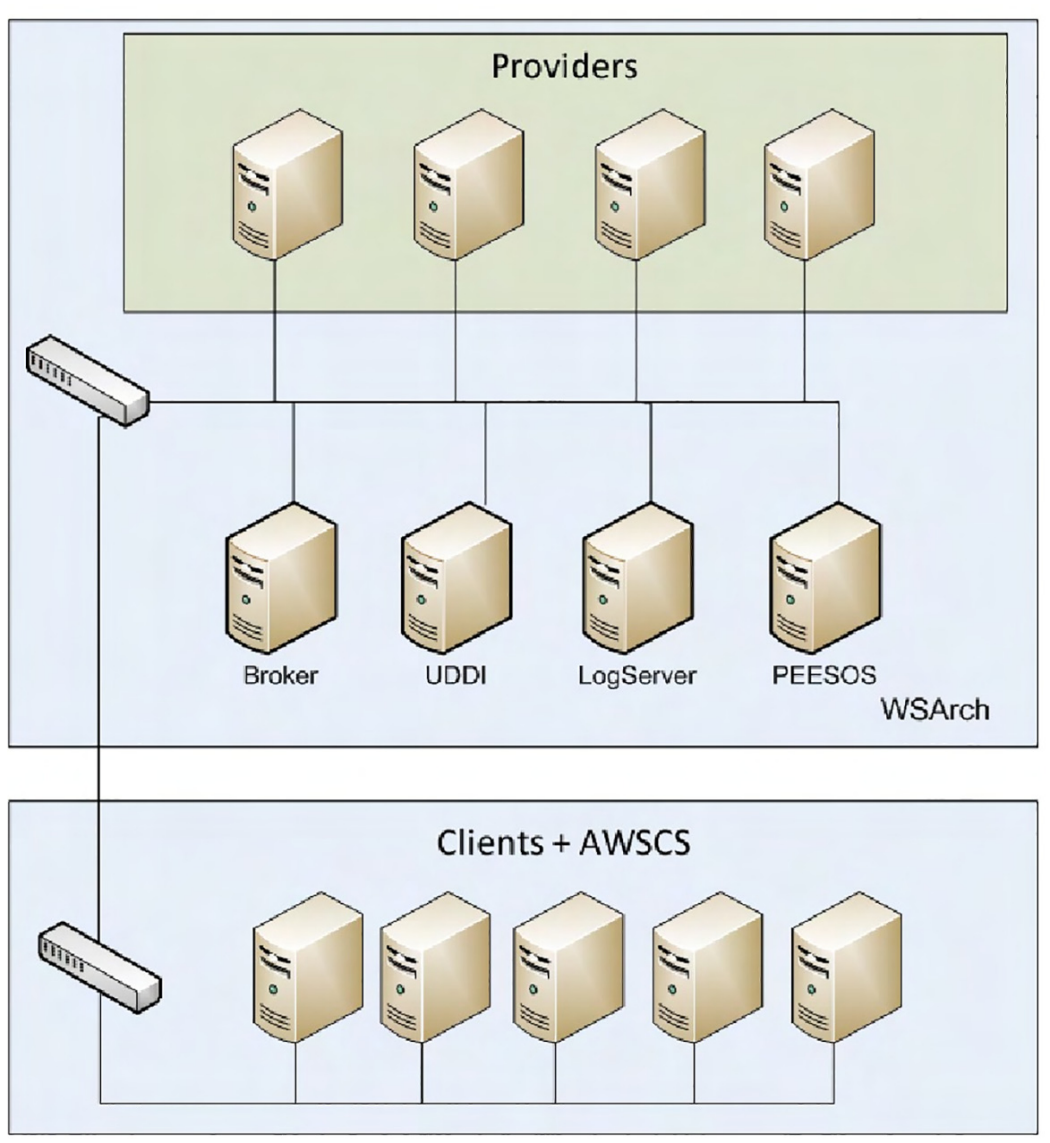
Experiments Environment.

In Fig 4 an overview is presented, containing the number of machines used, and how they are interconnected. The entire environment uses a gigabit network.

In the WSArch cluster presented in Table 1, four physical machines were used for hosting providers, in which each physical machine has three virtual machines, and each virtual machine hosts a provider of Web services, generating a total of 12 Web services providers. Each of the virtual machines has a dedicated gigabit network adapter to avoid package collision during the experiments. The Broker, UDDI, LogServer and PEESOS was hosted on a real machine.

In the second cluster, presented in Table 2, the clients and also the system AWSCS were hosted. For this experiment 5 machines with the AWSCS were used.

### 3.2 Selection Algorithm

The selection algorithm used in this experiments was the Euclidean Distance. However another approaches for QoS selection can be implemented in the WSArch Broker. The goal of the algorithm in this context is to find the Web service with QoS values closest as possible to the required value by the client [26]. The Euclidean distance is calculated by the Eq 1.

$$\begin{array}{c} \sqrt{{\left(x_{j}-x_{k}\right)}^{2}+{\left(y_{j}-y_{k}\right)}^{2}} \end{array}$$ (1)

It is important to point that this algorithm was used to select the concrete Web services for composition. Therefore, this algorithm has the purpose of providing QoS in execution and not achieve automatic composition of Web services. For providing QoS in the context of this project, the selection of Web services was performed according to the following criteria:

- For the Gold class, the algorithm aims to get the lowest response time. The goal is also to get the Web service with the best reputation possible. Since the cost is not a problem for this class, the algorithm tries to reach an average value. Thus the QoS attributes cost and response time are a priority for the Gold class.
- For the Silver class the algorithm aims to achieve all the goals achieving the average values for each QoS attribute.
- For the Bronze class the algorithm aims to achieve the minimum cost and the other attributes are already relaxed.

In the literature there are several proposals for Web services selection in the Web service composition context. Some examples of other proposals are presented in [27] [28] [29] [21], and these could easily be used in the proposed work as alternatives to the three level system.

### 3.3 Scenario

In Table 3, the SLAs contracts for the Web service composition flows used in our proposed scenario are presented. For each class a value is given that must not be exceeded for response time and cost and never be undercut for reputation. These SLA contracts are established according to the QoS offerings available for each Web service.

**Table 3:** SLAs Agreements.

**SLA**	**Response Time (ms)**			**Cost (US$)**			**Reputation (Stars)**		
	***Gold***	***Silver***	***Bronze***	***Gold***	***Silver***	***Bronze***	***Gold***	***Silver***	***Bronze***
**Hotel**	7000	10000	13000	200	130	60	3	2	1
**TripBus**	5250	6125	7000	40	35	30	3	2	1
**CityBus**	4250	5625	7000	3	3	3	3	2	1
**Event 1**	4500	5750	7000	53	31,5	10	3	2	1
**Event 2**	6500	7250	8000	30	25	20	3	2	1
**Event 3**	5000	6500	8000	70	45	20	3	2	1
**Night Event**	5000	6500	8000	70	45	20	3	2	1
**Roost**	6000	10000	14000	152	81	40	3	2	1
**Flight**	5400	6350	7300	120	110	100	3	2	1
**Taxi**	4250	5125	6000	30	330	30	3	2	1
**Event Packet**	6500	14250	22000	203	126,5	50	3	2	1

In the proposed scenario, two flows with the goal to propose a trip planning were developed. Both flows has the same semantic quality, since the aim of this paper is to evaluate the performance of the execution. The request considered for preparing the flow comprises the application for booking, transportation to the city of destination and back, transfers within the city and some events for entertainment.

**Fig 5:**
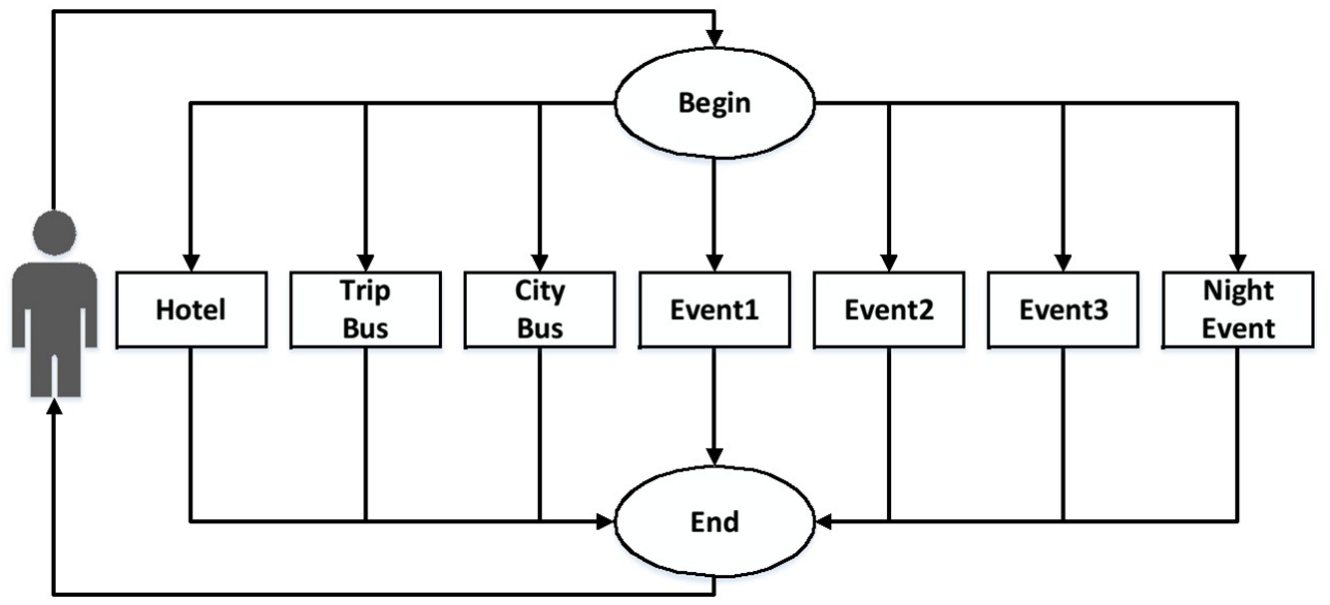
Flow 1.

Fig 5 shows flow 1. Flow 1 is formed by the Web service **Hotel** for hosting, **TripBus** regarding transportation to the destination city, **CityBus** for internal transfers and the Web services **Event1**, **Event2**, **Event3** and **NightEvent** for entertainment.

**Fig 6:**
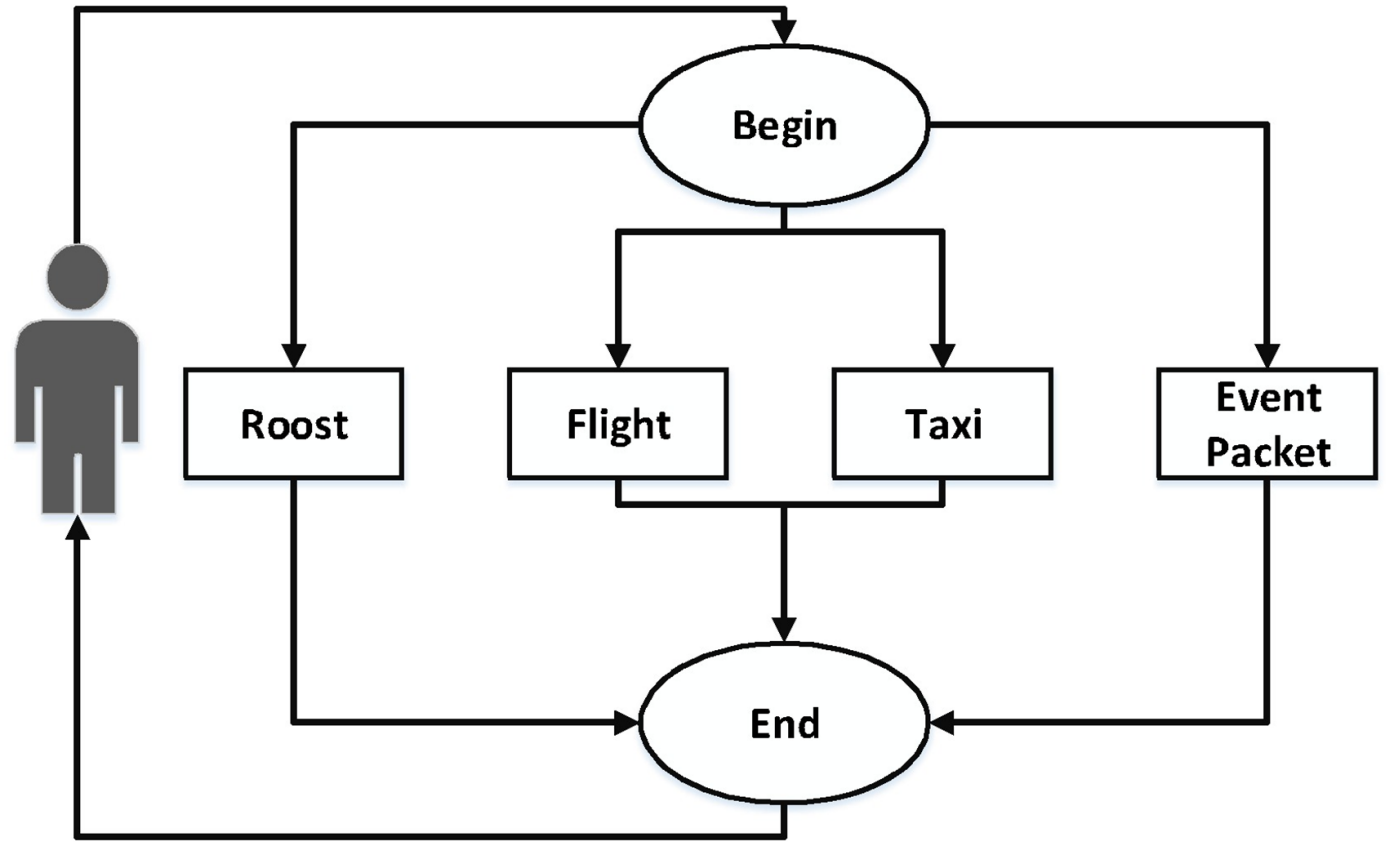
Flow 2.

Flow 2, shown in Fig 6, is composed of the Web services **Roost** for hosting service, **Flight** for the trip to the destination city, **Taxi** for internal transfers and the Web service **Event Packet**, a package of events responsible for providing entertainment to the client.

## 4 Results

In this section the results obtained by the execution of the experiments described in Section 3 are reported.

The experiments were performed following a full factorial combination and 10 replication for each possible configuration, resulting in 40 executions for each experiment. To calculate the confidence interval, a 95% confidence level was used. The load used for this experiment consists of 50 clients, the executions are controlled by PEESOS. The implementation follows an exponential distribution, also generated by PEESOS, to trigger the requests of these 50 clients.

### 4.1 Experiment 1

In the first experiment, we evaluate the behaviour of the implementation of the composite Web services, flow 1 and flow 2, in a scenario where only Gold class Web services were accessed. In Table 4 factors and levels used in the experiment are presented.

**Table 4:** Design of Experiments 1.

**Factor**	**Level 1**	**Level 2**
**AWSC algorithm**	Flow 1	Flow 2
**Workload**	Burst each 9s	Burst each 3s

In Table 5 the SLA contracts that must be respected during the experiment execution are described. Although all Web services run in parallel, the response time contract is set to 2 seconds longer than the highest Web service response time. This happens because the system needs some time to create the call and execute it against the Web service. Without that extra time, it would be impossible to meet the SLA in any condition of the system.

**Table 5:** Clients QoS restrictions.

**SLA**	**Flow 1**	**Flow 2**
**Response Time**	9000ms	8000ms
**Cost**	$ 466	$ 505
**Reputation**	3 Stars	3 Stars

**Fig 7:**
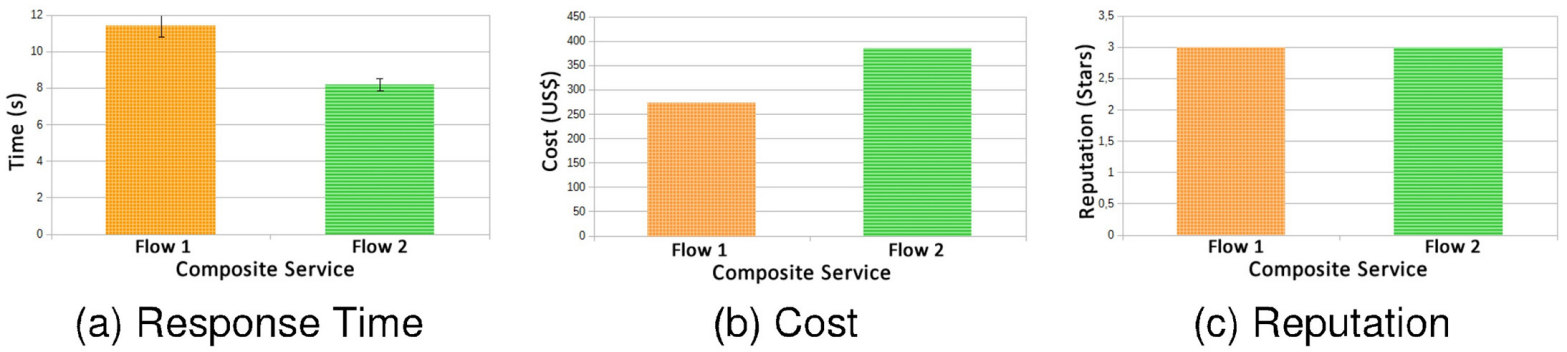
Flow 1 × Flow 2—bursts every 3000ms.

Fig 7 shows the Graphs related to the results of flows 1 and 2, where requests were executed in bursts every 3 seconds. For QoS attributes cost and reputation (graphs (b) and (c) respectively), there is no need to calculate the confidence interval, since these attributes are not associated with implementation and replication of the experiment execution, but are related with the choices of Web services that will be executed by the algorithm presented in Section 3.2.

In terms of reputation both flows have achieved the goal of the best possible. In terms of cost the first flow obtained considerably lower values, due to the types of Web services chosen, since in flow 1 the travel between cities is by bus, while in flow 2 is performed using plane. Also, flow 1 uses bus for local transport while flow 2 uses a taxi.

For the overload situation presented in the Graph (a) of Fig 7, the executions in bursts occur every 3 seconds. The flow 1 obtained a considerably worse performance compared to flow 2, since the average of the result surpasses the limit of the contract by 2 seconds. For flow 2, the average response time gets values a little above the limit from the SLA (which was defined as 8 seconds). Despite using different services in experiment 1 the flow 2 managed to be more efficient than flow 1, with the same load submitted to the system.

**Fig 8:**
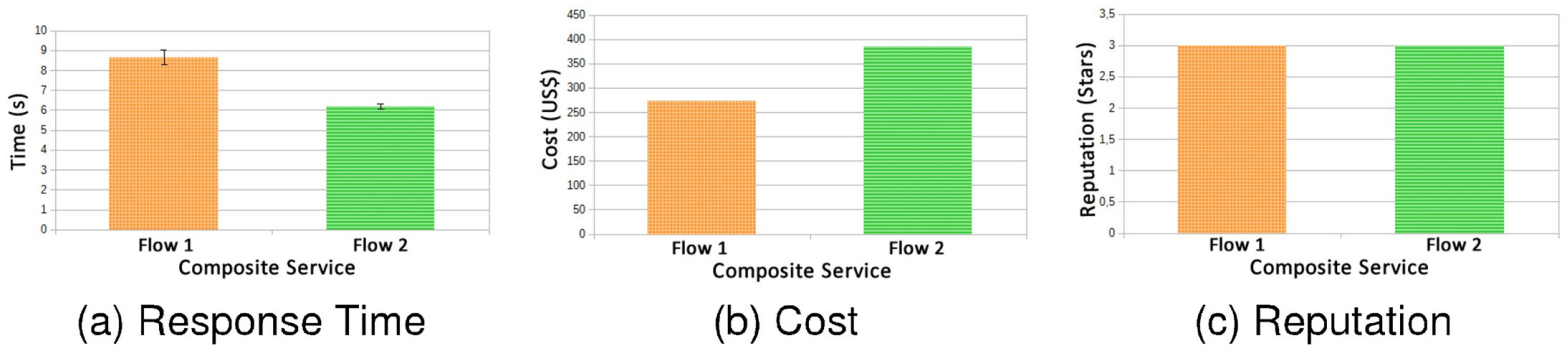
Flow 1 × Flow 2—bursts every 9000ms.

In Fig 8 the results for experiment 1 are presented, however, clients send requests in bursts each 9 seconds, slightly overloading the system. As the results obtained for the attributes of QoS cost and reputation are unaffected by the behaviour of execution, and in this case clients were all from Gold class, the results were repeated, as it is possible to observe in the Graphs (b) and (c) of Fig 8.

In the Graph (a) of Fig 8 in is possible to notice that flow 2 is able to maintain performance approximately 2 seconds below the SLA requirements fulfilling the contract for all requisitions. The flow 1, manages to in most times fulfil the SLA contract, however for some cases it did not happen, since the confidence interval got a little above 9 seconds.

**Fig 9:**
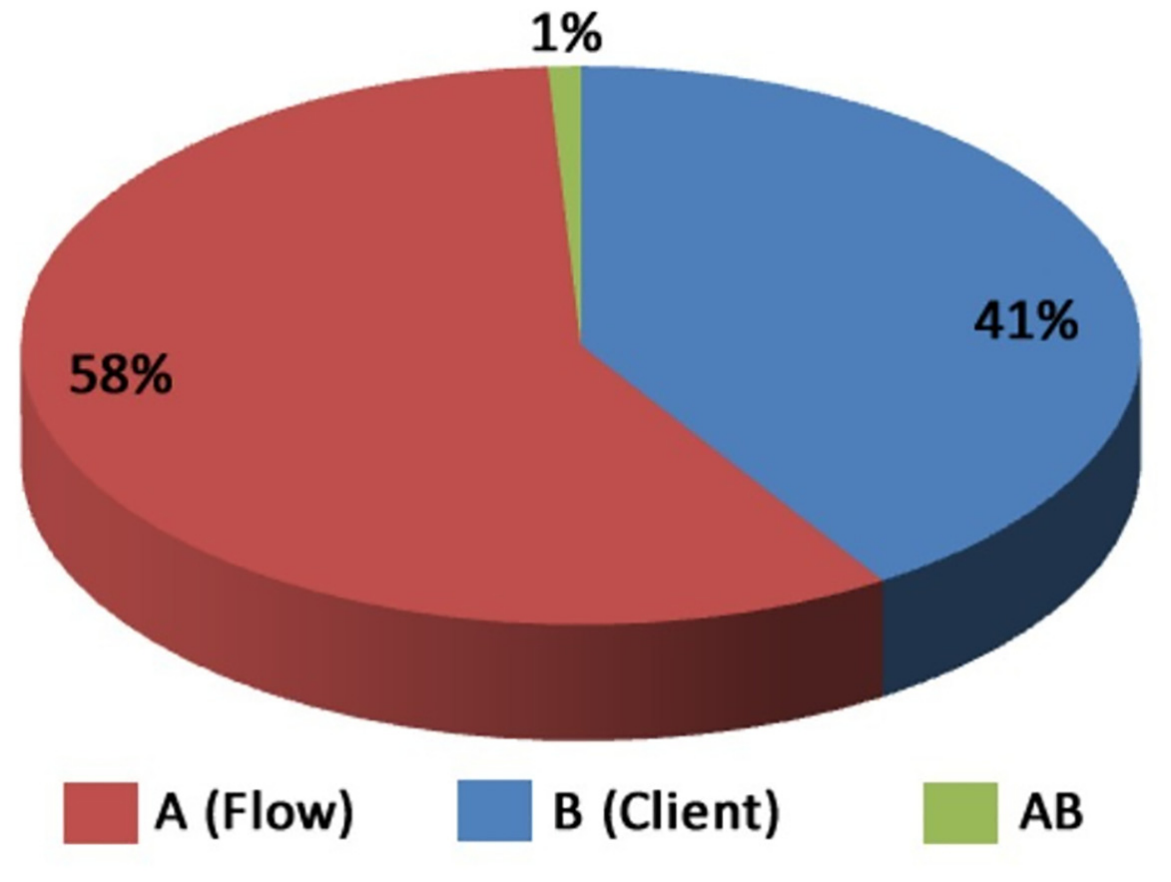
Flow 1 × Flow 2—Influence of Factors.

In Fig 9 the factors that most influenced the experiment are presented. As QoS attributes reputation and costs are fixed, it means that they do not vary with the execution, so it does not make sense to do this type of calculation for such factors (it always will result in 100% for the algorithm and 0% for the load). So in Fig 9 we present the influence of factors only for the response time. The calculations used in this paper to measure the factors effects was made based on the techniques presented in the book [30].

In the case of the first experiment, it is possible to note that the execution is critical to the effects of the flow used in response time, since the flow execution had 41% of effect on the results of this experiment.

### 4.2 Experiment 2

In experiment 2, we evaluated flows 1 and 2, considering a scenario in which the SLA was different for five types of clients. In Table 6 the classes for each Web service accessed are presented. In Table 7 the SLAs defined for each one of the five clients are presented.

**Table 6:** QoS Classes for each Web service and Client.

**Flow 1**					
**Client**	C1	C2	C3	C4	C5
**Hotel**	bronze	silver	gold	gold	bronze
**Citybus**	bronze	silver	gold	silver	bronze
**Tripbus**	bronze	silver	gold	silver	gold
**Event1**	bronze	silver	gold	gold	silver
**Event2**	gold	silver	gold	silver	silver
**Event3**	bronze	silver	bronze	bronze	silver
**NightEvent**	bronze	silver	gold	gold	silver
**Flow 2**					
**Client**	C1	C2	C3	C4	C5
**Roost**	bronze	silver	gold	gold	bronze
**Taxi**	bronze	silver	gold	silver	bronze
**FlightCompany**	bronze	silver	gold	silver	gold
**EventPacket**	silver	silver	gold	silver	silver

**Table 7:** SLA definition for each Client.

**Flow 1**					
**Client**	C1	C2	C3	C4	C5
**Response Time**	14000ms	12000ms	10000ms	10000ms	14000ms
**Cost**	173	314,5	416	406	239,5
**Reputation**	1	2	3	2	2
**Flow 2**					
**Client**	C1	C2	C3	C4	C5
**Response Time**	16000ms	12000ms	8000ms	12000ms	14000ms
**Cost**	260	437,5	505	418,5	296,5
**Reputation**	1	2	3	2	2

The factors are presented in Table 8.

**Table 8:** Factor and levels used in the experiment.

**Factor**	**Level 1**	**Level 2**	**Level 3**	**Level 4**	**Level 5**
**AWSC Algorithm**	Flow 1	Flow 2	-	-	-
**Load**	Bursts every 9s	Bursts every 1s	-	-	-
**Clients**	C1	C2	C3	C4	C5

**Fig 10:**
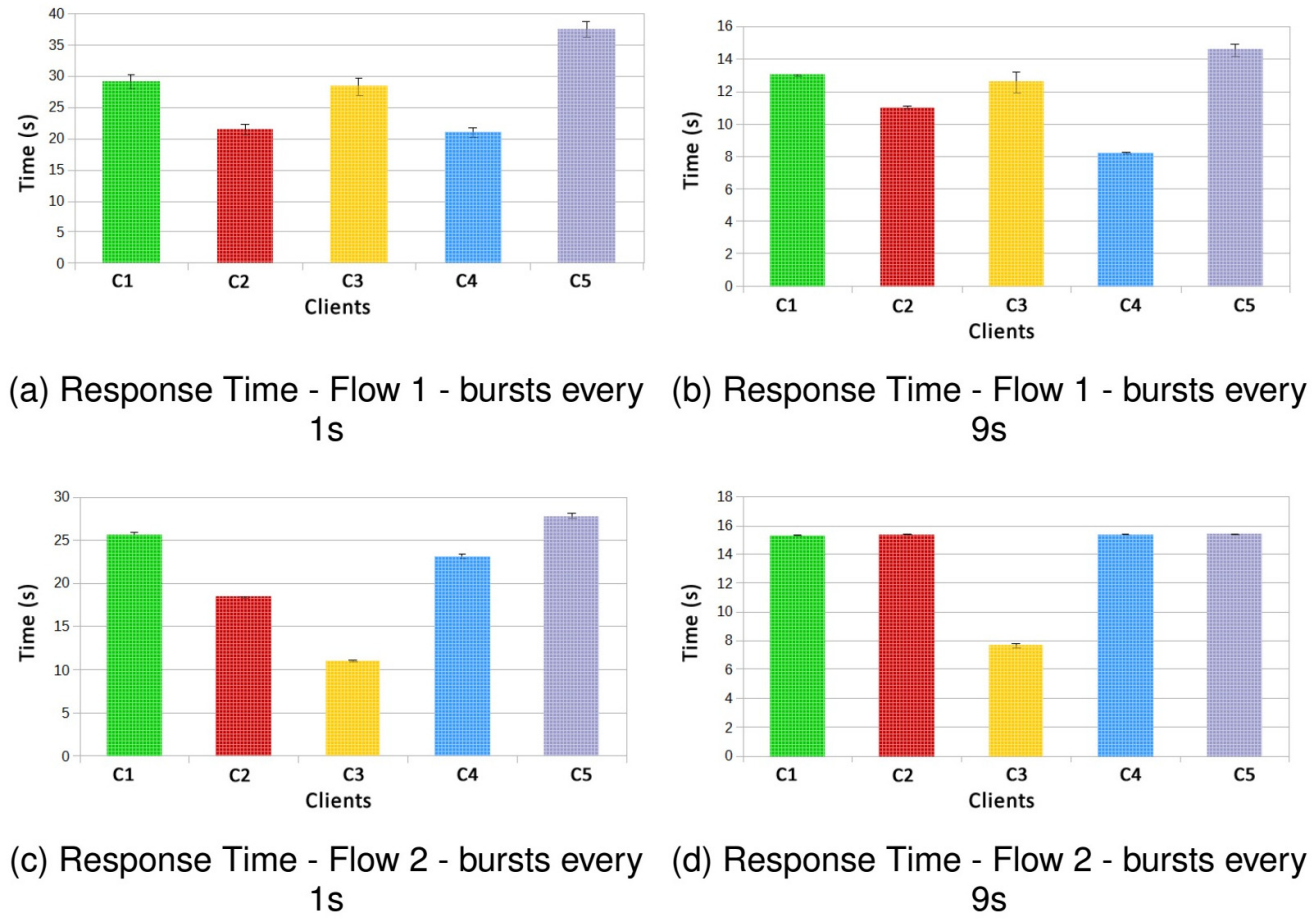
Response Time—Flow 1 × Flow 2—Different QoS for each Client.

In Fig 10 the results for the response time in the execution of flows 1 and 2 are shown. In cases where the bursts occur every 1 second, this entails a greater load on the system when compared with bursts triggered every 9 seconds. As in experiment 1, the C3 client that requests most Web services in the class Gold for both flows, performed better in the second flow. For other clients, all performs better in flow 1 than in flow 2 for low overload, and bursts every 9 seconds. This situation changes for high overload with bursts every 1 second, where all executions for all the clients from flow 2 perform better than those in flow 1.

Analysing SLAs for flow 1 (low overload), clients C1, C2 and C4 comply with the SLAs for response time. The client C3 was about 2 seconds above the SLA on average and client C5 was above some few tenths of a second with respect to the SLA on average. Whith a high overload, it is possible to observe that for flow 1, all clients do not respected the SLA and the delay in delivering the results varies greatly according to the SLA signed by each client.

Regarding the SLAs for flow 2 with low overload, only clients C1 and C3 can meet the SLA, with worse performance in this situation compared to flow 1. To high overload, the delay in response delivery is proportionally shorter than in the flow 1, and considering a scenario of high overload, flow 2 has better performance for the response time than flow 1.

**Fig 11:**
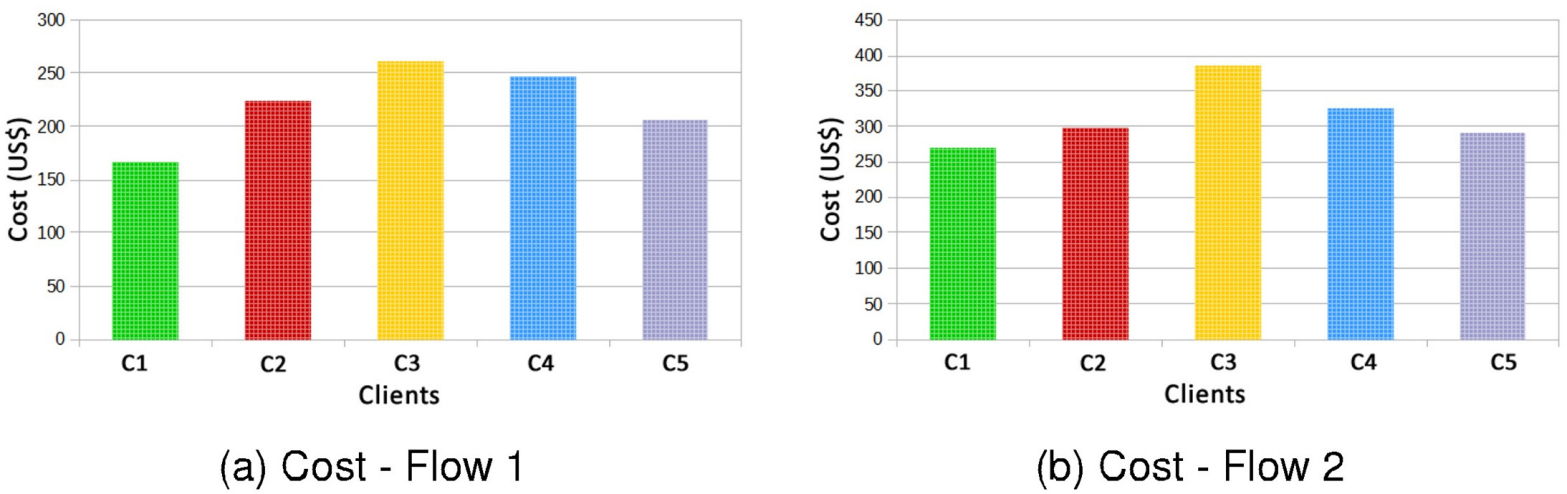
Cost Flow 1 × Flow 2—Different QoS for each Client.

In Fig 11 the costs for SLAs of Web services contracted by each of the five clients are presented. As in experiment 1, both two flows can meet the SLA contracts, and again, the flow 1 has the lowest cost in relation to flow 2.

**Fig 12:**
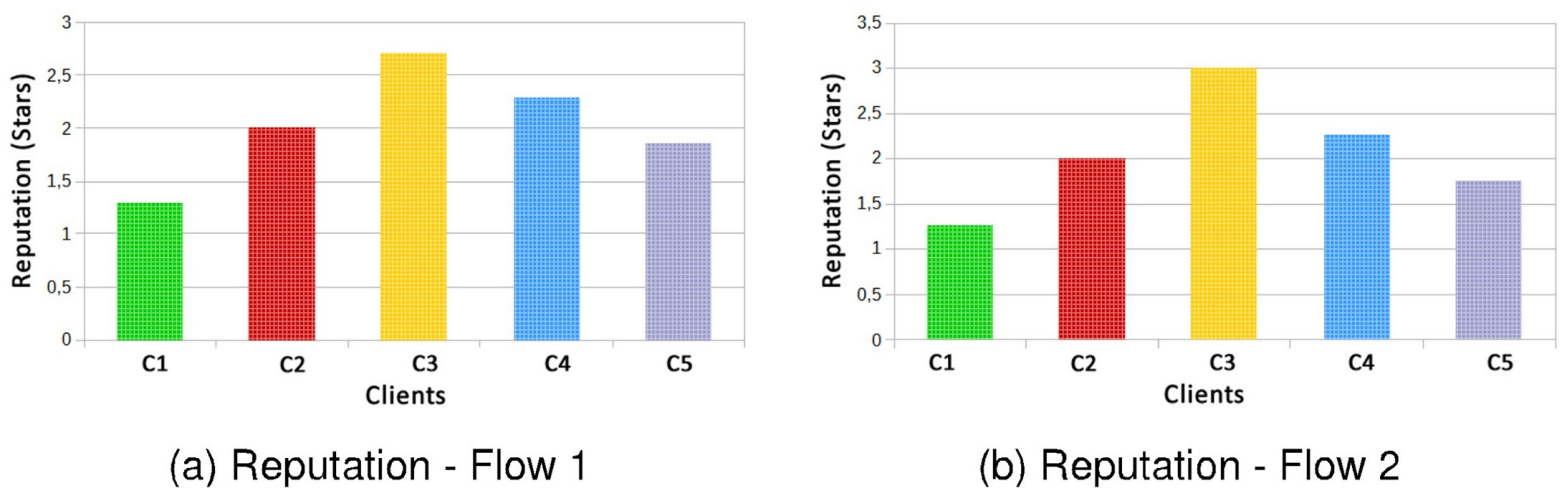
Reputation Flow 1 × Flow 2—Different SLA for each Client.

In Fig 12 reputations for SLAs of Web services contracted by each of five clients are presented. Regarding reputation it is possible to observe that in both flows, SLAs contracts are respected also achieving very similar values for both cases.

For experiment 2, it was very difficult to make the analysis of factors effects for the following reasons:

- There are 5 levels in the factor types of client, and the calculation of the factors effects is possible only isolating levels 2 by 2. Therefore it was decided to present only some combinations of the factors effects. The combinations used were (C1 to C2), (C1 to C3) and (C3 to C2).
- Since variation of the load is very high, in most calculations, it was the only factor which influenced the outcome of the experiment. Therefore we decide to isolate the load on the analysis of the influence of factors. So two results are presented for the influence of factors for each combination: one for low load and the other for high load.

**Fig 13:**
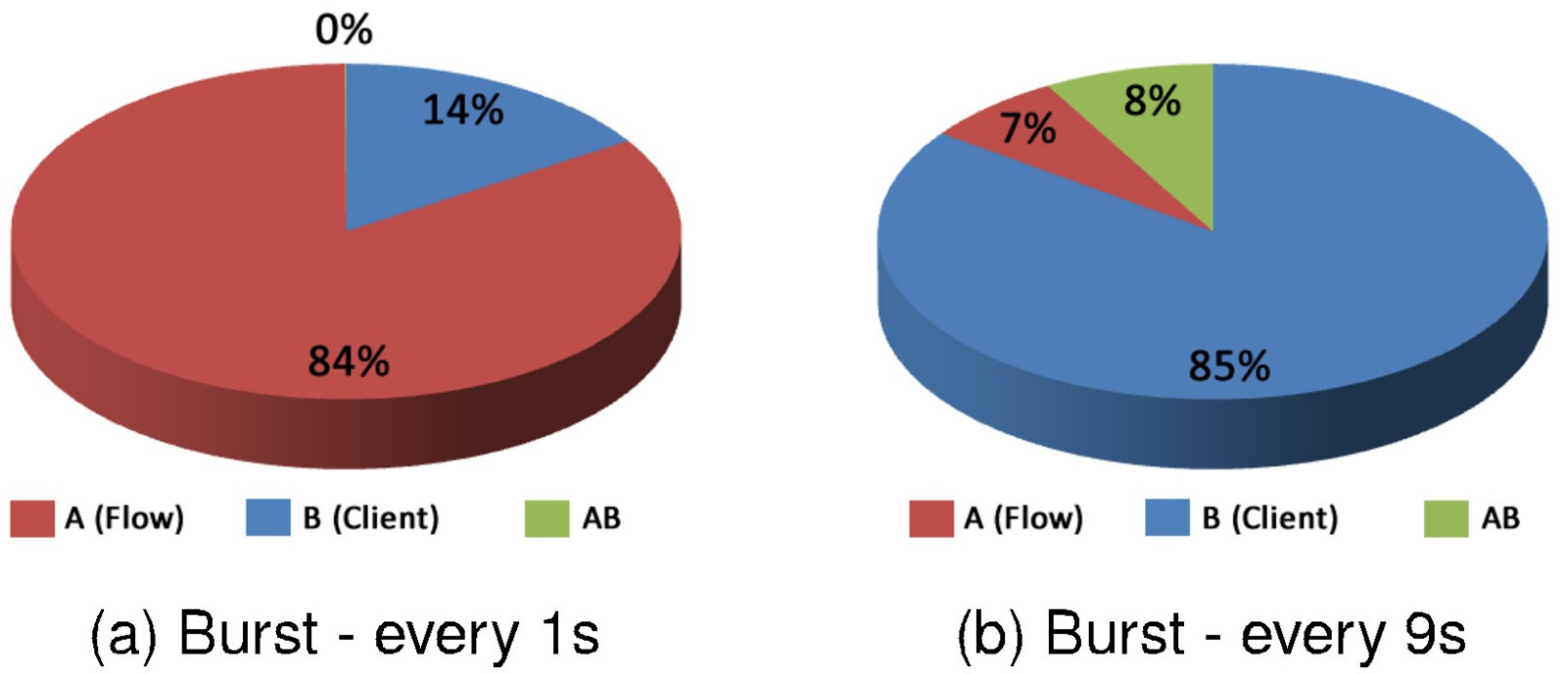
Factors influence—C1 with C2—Response Time.

In Fig 13 results are presented with the percentage that the factor flow and type of client have influenced the results. In Fig 13 the effect of client’s type C1 and C2 are calculated alone. In Graph (a) the results are presented where the requests were triggered in bursts every 1 second and (b) bursts every 9 seconds.

For the factor effects presented in the Graph (a) the factor type of client influenced 84% and the flow factor influenced 16%; the interaction between the two factors did not influence these results. In Graph (b) in a situation of low overload on the system, the scenario has completely reversed. In this case, the flow was the most important factor involved, reaching 85% of influence, while the factor type of client influenced 7%. The interaction of the two factors, influenced in 8% for this situation.

**Fig 14:**
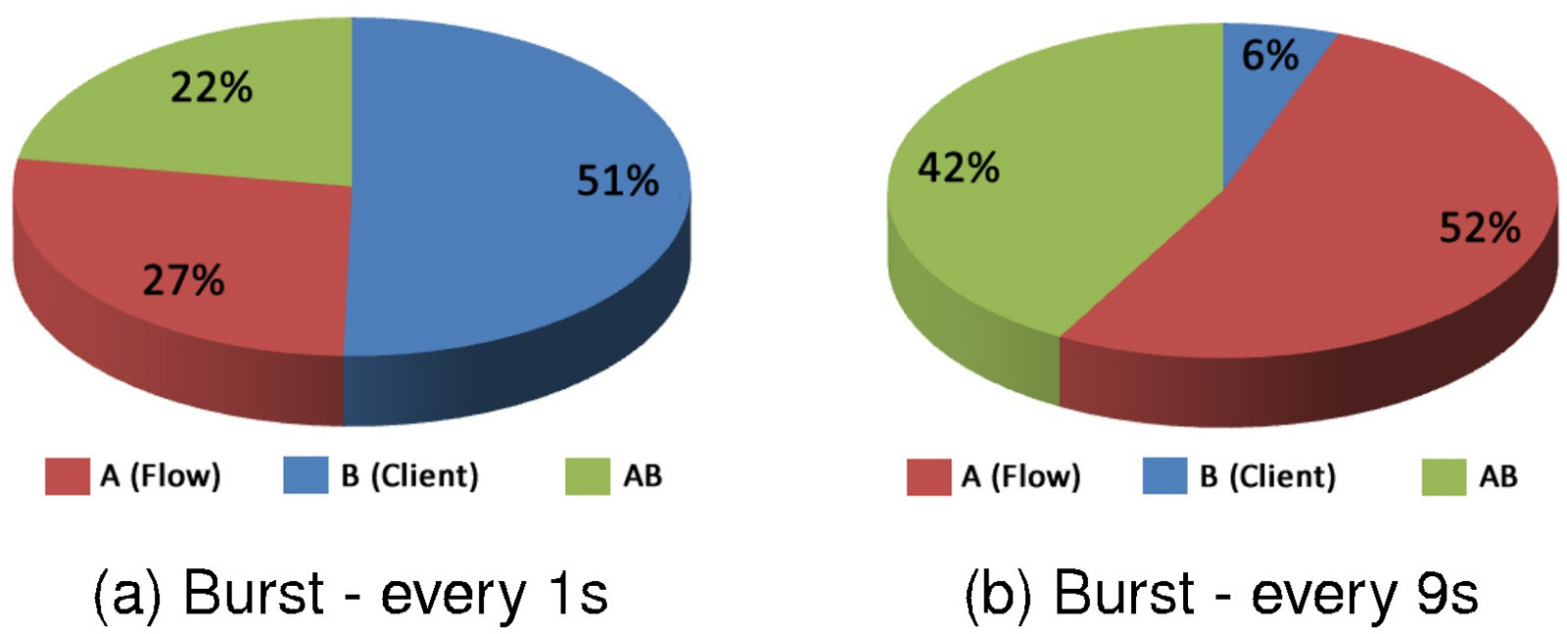
Factors influence—C1 with C3—Response Time.

In Fig 14 the influences of the factors for clients C1 and C3 are presented isolated. When analysing the influence of the factors for these clients, we observed that the factors effects had a different behaviour compared with the factor effects presented in Fig 13.

Graph (a) from the Fig 14 shows the factors effects for the high overload and it indicates that the flow factor has an influence of 51%, the client factor is 27% and the interaction between these two factors is 22%. In Graph (b) of Fig 14, the influence of the factors is reversed in this case, the type of client factor influenced in 52%, the flow factor influenced 6% and the interaction of factors influenced in 42%.

**Fig 15:**
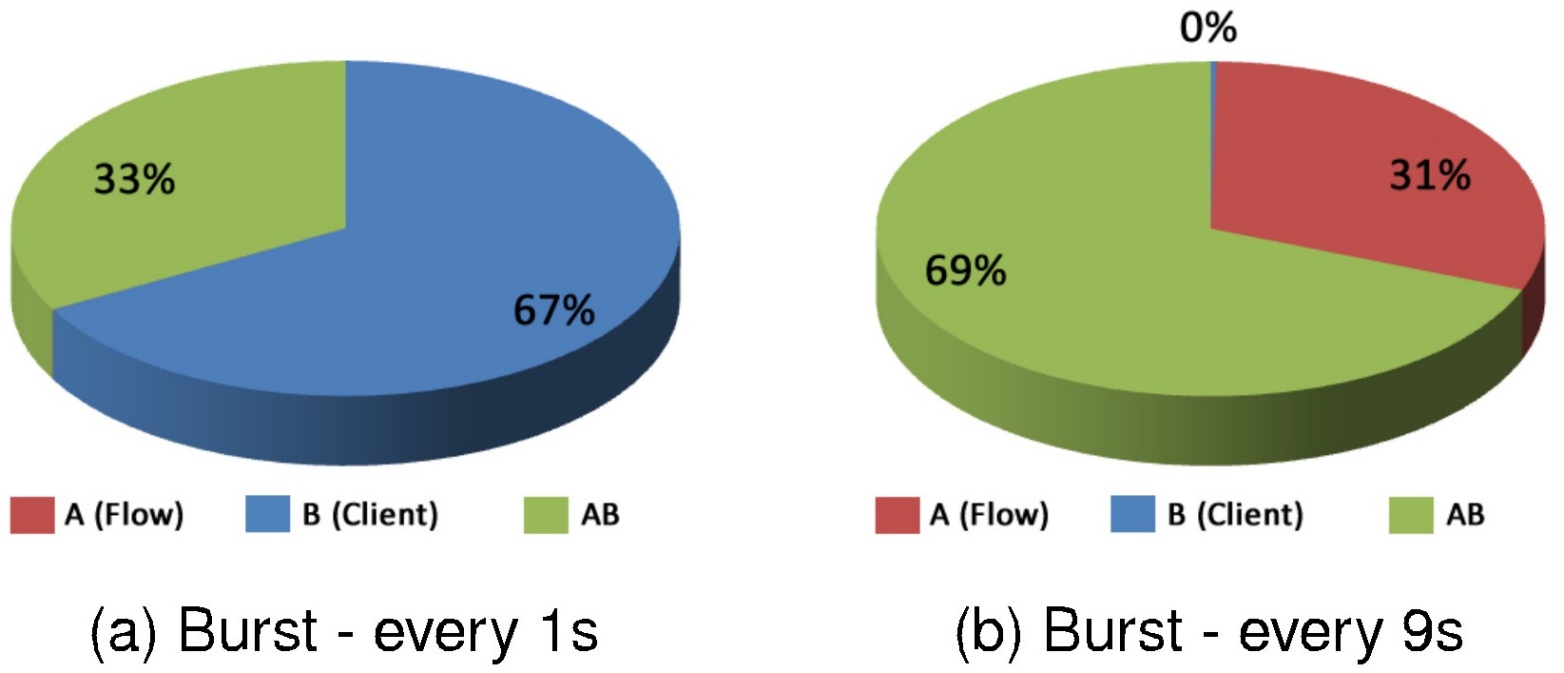
Factors influence—C2 with C3—Response Time.

In the Graph (a) of the Fig 15, where there is a high overload, the factor type of client influenced in 67%, the factor type of client influenced 0%, but the interaction of the two factors had an influence of 33%. In the Graph (b) the factor effects are presented in low overload. In this case the factor flow influenced 0%, the factor type of client influenced 31% in the results and the interaction of the two factors influenced 69%.

**Fig 16:**
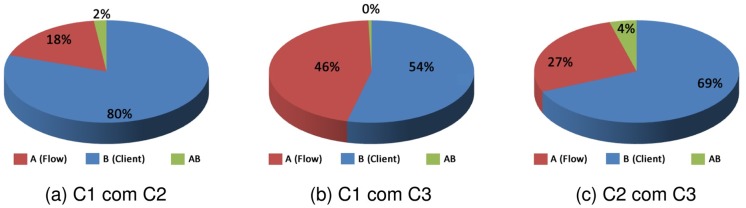
Factors Influence—Cost.

Fig 16 shows the influence of the factors for clients (a) C1 and C2, (b) C1 and C3, and (c) C2 and C3, for the response variable cost.

In Graph (a) of Fig 16, the influence of factors presented in these results suggests that the flow factor influenced by 80%, the client factor 18% and the interaction between the two factors 2%. In the Graph (b) of Fig 16, the flow factor influenced 54%, the client factor influenced 46% and the interaction of factors influenced in almost 0%. In Graph (c), again the factor that most influenced the result was the flow with 60%, the client factor influenced 27% and the interaction of factors influenced 4%.

**Fig 17:**
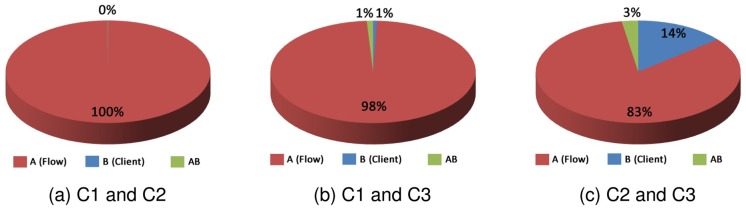
Factors Influence—Reputation.

In Fig 17 are shown the factor effects for clients (a) C1 and C2, (b) C1 and C3, and (c) C2 and C3, whereas the response variable reputation.

In Graph (a) of the Fig 17, the effect for the type of client factor influenced in almost 100%, and the client and the interaction between the two factors is almost 0%. In Graph (b) of the Fig 17, the client factor influenced by 98% on the result, the flow factor influenced 1% and the interaction of factors influenced 1%. In Graph (c), the factor that most influenced the result was the type of client with 83%, the flow factor influenced 14% and the interaction of factors influenced 3%.

## 5 Conclusion

A good choice of approach for Web services composition can be a crucial issue for better user satisfaction, thus increasing the chances of success in using Automatic Composition of Web services. The choice may vary according to the environment where it will be implemented, so it is important to have a system where you can test the possible approaches for Web services composition before putting them into production.

In this paper we proposed a system named AWSCS in which is possible to evaluate approaches for automatic composition of Web services based on QoS parameters that depend on the execution to be measured.

In the experiments presented in this paper, we demonstrated how unpredictable is the behaviour of executing composite Web services. This is especially true when using a scenario where the load, composition type and client type undergoes changes.

The prototype presented in this paper is a functional version of AWSCS, however there is still much work to be done to progress the AWSCS to make it even more user-friendly tool.

Among the proposed improvements for future work some can be highlighted. For example, the implementation of algorithms for automatic composition of Web services, having the system a complete environment for automatic composition of Web services, from planning the flow of composition to executing the flow. But most crucially we will provide the opportunity to plug composition algorithms into the tool, allowing users to compare the compositions their tools achieve.

Another improvement is to create a repository of Web services. There is a project named OWLS-TC [31], that offers a collection of semantic definitions for testing, however, implementations of Web services do not exist. Therefore, there is a need to create a repository where, besides the semantic definitions, there are implementations for testing Web services.

With the fault tolerance system proposed in [32]. Integrated with the *Broker* system, it will be possible to measure the impact of the failure to access the Web services running on the composite Web service.

Finally, we are planning to implement security policies in the system and evaluate the impacts of these policies on Web services. The development of AWSCS system did not contemplate any aspect of safety in the first version of its prototype. This is a very important consideration, given that Web services are often executed using the Internet as infrastructure.
